# The Impact of Empathy and Coping Traits on Psychological Responses: A Longitudinal Study on Medical and Nursing Students

**DOI:** 10.1007/s10880-026-10144-1

**Published:** 2026-03-10

**Authors:** Taku Saito, Norihito Noguchi, Kotaro Shoji, Fumiko Waki, Masanori Nagamine

**Affiliations:** 1 Division of Behavioral Science, National Defense Medical College Research Institute, National Defense Medical College, Tokorozawa, Japan; 2 College of Nursing, Osaka Dental University, Hirakata, Japan; 3 College of Nursing, University of Human Environments, Okazaki, Japan

**Keywords:** Empathy, Coping, Medical and nursing students, Secondary traumatic stress, Burnout, Compassion satisfaction, Latent profile analysis

## Abstract

**Introduction:**

Healthcare professionals are expected and motivated to engage empathetically with patients, their families, yet how different components of empathy, coping traits interact to shape psychological responses remains unclear. This study examined these relationships in medical, nursing students to inform tailored educational interventions

**Methods:**

Participants who completed two surveys 2 years apart (30 medical students and 88 nursing students) were included. Empathy traits were assessed using the Interpersonal Reactivity Index, coping traits using the Brief Coping Orientation to Problems Experienced Inventory, and psychological responses using the Professional Quality of Life Scale. Mixed-effects models for repeated measures analyzed the impact of empathy and coping traits. Latent profile analysis (LPA) classified participants by empathy and coping traits.

**Results:**

Personal distress was significantly associated with increased secondary traumatic stress (STS) and burnout, and decreased compassion satisfaction. Empathic concern was associated with increased compassion satisfaction. Active coping and support seeking were associated with increased compassion satisfaction and reduced burnout, whereas indirect coping was associated with increased STS and burnout. LPA identified three distinct profiles of empathy and coping traits, showing significant differences in psychological responses.

**Discussion:**

Differences in empathy and coping traits influence psychological responses in medical and nursing students. Tailored interventions that consider these traits may be more effective.

**Supplementary Information:**

The online version contains supplementary material available at https://doi.org/10.1007/s10880-026-10144-1.

## Introduction

Healthcare professionals are expected and motivated to engage empathetically with patients and their families, including situations involving patient death and emotional distress. This emotional involvement can lead to negative psychological responses, such as emotional exhaustion, burnout (Freudenberger, 1974), and post-traumatic stress presenting as compassion fatigue or secondary traumatic stress (STS) (Figley, 1995, 2002).

Empathy is conceptualized into cognitive empathy, understanding others’ internal states, and emotional empathy, resonating emotionally with others (Davis, 2006). Traditionally, detached concern—a stance of maintaining cognitive empathy while keeping emotional distance—was recommended for healthcare professionals (Fox & Lief, 1963). However, since the 1990s, studies have reported that fully expressing both cognitive and emotional empathy enhances patient compliance, improves treatment outcomes, and increases satisfaction for both patients and healthcare professionals (Derksen et al., 2013). Consequently, in recent years, the concept of detached concern has been increasingly replaced by the predominant view that healthcare professionals should fully engage in both cognitive and emotional empathy when interacting with patients (Halpern, 2007). Further, numerous educational interventions have aimed to enhance empathy (Kelm et al., 2014).

However, prior research on physicians indicates that empathic concern—compassion and concern for others’ suffering—positively correlates with compassion satisfaction but also with burnout and STS (Gleichgerrcht & Decety, 2013). Notably, personal distress, characterized by anxiety when confronting others’ suffering, shows a negative correlation with compassion satisfaction and a positive correlation with burnout and STS, making it a high-risk empathy trait linked to psychopathology (Gleichgerrcht & Decety, 2013). A cross-sectional study of approximately 1,000 public health nurses identified a high personal distress and STS group through latent profile analysis (Kitano et al., 2023).

These findings suggest the existence of a vulnerable group of healthcare professionals who are prone to negative psychological responses due to specific empathic traits, emphasizing the need for empathy training that incorporates coping strategies and considers individual empathy traits, rather than merely teaching healthcare professionals how to be more empathetic. However, previous studies have rarely focused on clarifying the direct relationship between components of empathy and negative psychological outcomes, and longitudinal investigations into the mechanisms and effects of this relationship remain insufficient.

Therefore, this longitudinal study aims to explore the previously underexamined issue of how components of empathy and coping traits influence psychological responses in medical and nursing students both during their clinical practice phase and into their actual clinical engagement. The goal is to provide new insights for medical education and facilitate the development of tailored interventions for empathy and coping training.

## Methods

### Study Design and Participants

The participants of this prospective cohort study were medical and nursing students at the National Defense Medical College who were engaged in clinical practice. A self-administered questionnaire was given to 150 fifth-year medical students and 240 third-year nursing students at Time 1 (March to May 2020 or March to May 2021), after participants had had approximately one year of clinical practice. A follow-up survey was administered 2 years later at Time 2 (March to May 2022 or March to May 2023), when they had graduated from the college and had been engaged in practical clinical work as residents or nurses in the hospital for about a year. Participants who completed both surveys were included in the analysis. Written informed consent was obtained from all participants. The study was conducted in accordance with the Declaration of Helsinki and approved by the Ethics Committee of the National Defense Medical College (Approval No. 4040).

### Measures

Participants were assessed using self-administered questionnaires and reported their basic demographic information such as age and sex.

### Japanese Version of the Interpersonal Reactivity Index (IRI)

The IRI is a self-administered questionnaire comprising 28 items designed to evaluate empathic traits. It is divided into four subscales: empathic concern, perspective taking, personal distress, and fantasy (Davis, 1980; Himichi et al., 2017). Empathic concern measures “the extent of sympathy and concern for others,” perspective taking evaluates “the ability to adopt another person’s viewpoint,” personal distress assesses “the degree of anxiety and fear induced by others’ distress,” and fantasy refers to “the tendency to imaginatively place oneself in others’ situations” (Davis, 1980; Himichi et al., 2017). Empathic concern and personal distress reflect emotional aspects of empathy, while perspective taking and fantasy reflect cognitive aspects (Davis, 1980; Himichi et al., 2017). All items are scored on a 5-point Likert scale, ranging from 1 (does not describe me well) to 5 (describes me very well), with higher scores indicating stronger empathic traits. The Cronbach’s α for empathic concern, perspective taking, personal distress, and fantasy at Time 1 were 0.77, 0.75, 0.79, and 0.84, respectively, and 0.80, 0.77, 0.81, and 0.85 at Time 2.

### Japanese Version of the Professional Quality of Life Scale (ProQOL)

The ProQOL is a 30-item self-administered questionnaire that evaluates psychological responses and consists of three subscales: compassion satisfaction, burnout, and STS (Fukumori et al., 2018; Stamm, 2013). Each item is rated on a 5-point Likert scale, ranging from 1 (never) to 5 (very often), with higher scores indicating stronger psychological responses. The Cronbach’s α values for compassion satisfaction, burnout, and STS at Time 1 were 0.86, 0.71, and 0.70, respectively, and 0.93, 0.81, and 0.76 at Time 2.

### Japanese Version of the Brief Version of the Coping Orientation to Problems Experienced Inventory (Brief COPE)

The Brief COPE is a 28-item self-administered questionnaire that assesses coping strategies individuals use when facing problems (Carver, 1997; Otsuka, 2009). All items are rated on a 4-point Likert scale from 1 (I have not been doing this at all) to 4 (I have been doing this a lot), with higher scores indicating better coping strategies. Based on previous research (Shoji et al., 2024), we used three of this inventory’s subscales in this study: active coping, support seeking, and indirect coping. The Cronbach’s α values for active coping, support seeking, and indirect coping at Time 1 were 0.84, 0.79, and 0.74, respectively, and 0.84, 0.80, and 0.74 at Time 2.

### Statistical Analysis

There were 15 responses (0.07% of the total) with missing data on age, Brief COPE, or ProQOL items. The results of the Missing Completely at Random (MCAR) test (age: *χ*^*2*^(2) = 5.53, *p* = .063; Brief COPE: *χ*^*2*^(27) = 20.9, *p* = .793; ProQOL: *χ*^*2*^(29) = 16.3, *p* = .972) indicated that all missing values were random. Therefore, missing data were imputed using the missForest R package (Stekhoven & Bühlmann, 2012). Chi-square tests or Fisher’s exact tests were used to compare participants’ demographic characteristics, and *t*-tests were conducted to compare the mean scores of each scale. Pearson’s correlation coefficients were calculated among scale scores at Time 1 and Time 2.

To evaluate the impact of empathy and coping traits on longitudinal changes in psychological responses from Time 1 to Time 2, mixed-effects models for repeated measures (MMRM) were conducted using the lme4 package in R (Bates et al., 2015). In the first model, fixed effects included time, gender, student type (medical or nursing student), the four subscales of the IRI, and the three subscales of the Brief COPE. Next, latent profile analysis (LPA) was performed using standardized scores of the IRI and Brief COPE subscales to classify participants based on their empathy and coping traits. The optimal model was selected based on the largest Bayesian Information Criterion (BIC). Continuous variables were compared across the latent profiles using one-way analysis of variance (ANOVA). Based on the identified profiles, a second MMRM was conducted. In this model, fixed effects included time, gender, student type, and the latent profiles derived from LPA. In both models, random intercepts were specified at the individual level, and all variables were centered prior to the analysis. Model fit was assessed using Marginal *R*^*2*^ and Conditional *R*^*2*^ (Nakagawa & Schielzeth, 2013). All statistical analyses were conducted using R (version 4.3.0) (R Core Team, 2023) and RStudio (version 2023.06.0) (RStudio Team, 2023). Statistical significance was defined as *p* < .05.

## Results

Among the study participants (150 fifth-year medical students and 240 third-year nursing students), responses at Time 1 were obtained from 63 medical students and 139 nursing students. Of these, responses at Time 2 were obtained from 30 medical students and 88 nursing students, who were then included in the analysis. Participants who only responded at Time 1 (33 medical students and 51 nursing students) were excluded. Table 1 presents the participants’ demographic characteristics and scale scores between Time 1 and Time 2. The ratio of female to male participants significantly differed between medical and nursing students (*χ*^2^(1) = 38.7, p < .001). Empathic concern significantly decreased in the total sample and among medical students. In contrast, personal distress significantly increased in the total sample and among nursing students. Active coping significantly decreased in the total sample, while support seeking significantly increased both in the total sample and among nursing students. Compassion satisfaction significantly decreased in the total sample, as well as among medical and nursing students. Burnout significantly increased in the total sample, with a similar pattern observed in both medical and nursing students. Supplementary Table 1 presents the demographic characteristics of the included and excluded participants. No significant differences were found in demographic characteristics or scale scores between the included and excluded participants for both medical and nursing students. Supplementary Table 2 presents Pearson’s correlation coefficients among scale scores at Time 1 and Time 2. At both times, personal distress was positively correlated with STS (Time 1: *r* = .32; Time 2: *r* = .54) and burnout (Time 1: *r* = .37; Time 2: *r* = .49), while active coping was positively correlated with compassion satisfaction (Time 1: r = .42; Time 2: r = .54).

**Table 1:** Participants’ demographic characteristics and average scores for each scale between Time 1 and 2

Participants	Sex (men: female)	Age (mean, SD)	Time	Interpersonal Reactivity Index (mean, SD)				Brief COPE (mean, SD)			ProQOL (mean, SD)		
				Personal distress	Empathic concern	Perspective taking	Fantasy tendency	Active coping	Support seeking	Indirect coping	Secondary traumatic stress	Compassion satisfaction	Burnout
Total n = 118	22:96	21.9 (1.4)	1	21.9 (5.2)	24.4 (4.4)	22.2 (4.8)	23.6 (5.9)	23.7 (3.6)	16.9 (3.8)	28.3 (5.0)	18.3 (4.7)	29.6 (6.4)	26.7 (5.4)
			2	23.0 (5.2)	23.6 (4.9)	22.6 (4.7)	23.5 (6.0)	23.0 (3.9)	17.7 (3.5)	28.0 (4.8)	19.0 (4.9)	27.3 (7.4)	29.6 (6.1)
			Test statistic	3.1	2.4	1.2	0.4	2.1	2.5	0.7	1.7	3.8	6.5
			*p* value	0.003	0.019	0.25	0.67	0.035	0.015	0.46	0.087	< 0.001	< 0.001
Medical Student n = 30	15:15	23.9 (0.9)	1	23.0 (6.8)	23.1 (5.7)	21.3 (5.9)	23.8 (7.0)	23.5 (3.3)	15.6 (3.4)	29.1 (5.1)	17.2 (4.6)	30.2 (6.3)	26.0 (5.5)
			2	22.5 (6.8)	21.7 (6.3)	21.9 (5.8)	21.9 (7.2)	22.9 (4.1)	16.4 (4.4)	29.0 (5.2)	18.8 (5.8)	27.5 (7.3)	29.6 (6.9)
			Test statistic	0.8	2.2	0.8	2.5	1.0	1.4	0.05	1.9	2.4	4.1
			*p* value	0.45	0.033	0.42	0.018	0.32	0.16	0.96	0.065	0.023	< 0.001
Nursing Student n = 88	7:81	21.2 (0.8)	1	21.6 (4.5)	24.8 (3.8)	22.5 (4.4)	23.6 (5.5)	23.7 (3.7)	17.4 (3.9)	28.1 (5.0)	18.7 (4.6)	29.4 (6.4)	26.9 (5.4)
			2	23.2 (4.6)	24.3 (4.1)	22.8 (4.3)	24.0 (5.4)	23.1 (3.9)	18.1 (3.1)	27.7 (4.6)	19.1 (4.6)	27.3 (7.4)	29.7 (5.8)
			Test statistic	4.2	1.5	0.8	1.0	1.9	2.0	0.8	0.9	3.0	5.2
			*p* value	< 0.001	0.15	0.40	0.31	0.064	0.046	0.44	0.40	0.003	< 0.001

*Brief COPE*, Brief Version of the Coping Orientation to Problems Experienced inventory; *ProQOL*, Professional Quality of Life scale

The results of the MMRM with STS, burnout, and compassion satisfaction as dependent variables are shown in Table 2. For STS, significant positive main effects were observed for time, personal distress, and indirect coping (*t* = 2.3, *p* = .025; *t* = 4.5, *p* < .001; *t* = 4.0, *p* < .001). For burnout, significant positive main effects were found for time, personal distress, and indirect coping (*t* = 3.8, *p* < .001; *t* = 5.3, *p* < .001; *t* = 3.5, *p* < .001), while significant negative main effects were found for empathic concern, active coping, and support seeking (*t* = -2.1, *p* = .033; *t* = −4.6, *p* < .001; *t* = -2.0, *p* = .042). For compassion satisfaction, significant positive main effects were observed for empathic concern and active coping (*t* = 2.7, *p* = .008; *t* = 5.7, *p* < .001), while a significant negative main effect was found for personal distress (*t* = −2.7, *p* = .008). Additionally, a significant interaction effect was found between time and personal distress (*t* = 2.4, *p* = .020). Variance inflation factors (VIFs) were examined to assess multicollinearity among predictors, including gender and student type. All VIF values were below 5, indicating no problematic multicollinearity in the models. In the MMRM with STS, burnout, and compassion satisfaction as the dependent variables, the Conditional *R*^*2*^ showed an improvement over the Marginal *R*^*2*^. Supplementary Fig. 1 illustrates changes in compassion satisfaction from Time 1 to Time 2 across three groups categorized by personal distress levels (high: top 25%, moderate: middle 50%, low: bottom 25%). The decline in compassion satisfaction from Time 1 to Time 2 was steeper in the low and moderate groups than in the high group.

**Table 2:** Mixed-effects models for repeated measures with psychological responses as dependent variables

Dependent variables	Secondary traumatic stress			Burnout			Compassion satisfaction		
Independent variables	Estimate	*t* value	*p* value	Estimate	*t* value	*p* value	Estimate	*t* value	*p* value
Time	1.91	2.27	0.025	3.58	3.78	< 0.001	1.85	1.65	0.10
Female (ref: Male)	1.15	1.12	0.27	0.06	0.06	0.96	2.34	1.63	0.11
Nursing student (ref: Medical student)	0.69	0.80	0.42	1.56	1.64	0.10	2.91	2.36	0.020
Interpersonal Reactivity Index									
Personal distress	0.29	4.48	< 0.001	0.38	5.28	< 0.001	0.24	2.67	0.008
Empathic concern	0.06	0.80	0.43	0.19	2.14	0.033	0.30	2.66	0.008
Perspective taking	0.04	0.57	0.57	0.10	1.20	0.23	0.12	1.19	0.24
Fantasy tendency	0.04	0.63	0.53	0.06	0.93	0.35	0.06	0.66	0.51
Brief COPE									
Active coping	0.05	0.63	0.53	0.44	4.58	< 0.001	0.68	5.69	< 0.001
Support seeking	0.06	0.67	0.50	0.20	2.05	0.042	0.22	1.81	0.07
Indirect coping	0.25	4.01	< 0.001	0.24	3.53	< 0.001	0.10	1.19	0.23
Interaction									
Time*Personal distress	0.04	0.38	0.70	0.07	0.60	0.55	0.31	2.37	0.020
Time*Empathic concern	0.07	0.55	0.59	0.03	0.25	0.80	0.03	0.16	0.88
Time*Perspective taking	0.03	0.30	0.76	0.006	0.05	0.96	0.01	0.09	0.93
Time*Fantasy tendency	0.08	0.87	0.38	0.06	0.55	0.58	0.12	1.04	0.30
Time*Active coping	0.02	0.13	0.90	0.11	0.69	0.50	0.20	1.06	0.29
Time*Support seeking	0.09	0.69	0.50	0.01	0.08	0.93	0.13	0.70	0.48
Time*Indirect coping	0.12	1.23	0.22	0.05	0.46	0.64	0.16	1.15	0.25
Time*Nursing student (ref: Medical student)	1.79	1.83	0.070	1.72	1.56	0.12	0.57	0.44	0.66
Fit Index									
Marginal *R*^2^	0.29			0.42			0.34		
Conditional *R*^2^	0.61			0.67			0.68		

*Brief COPE* brief version of the coping orientation to problems experienced inventory

**Fig. 1:**
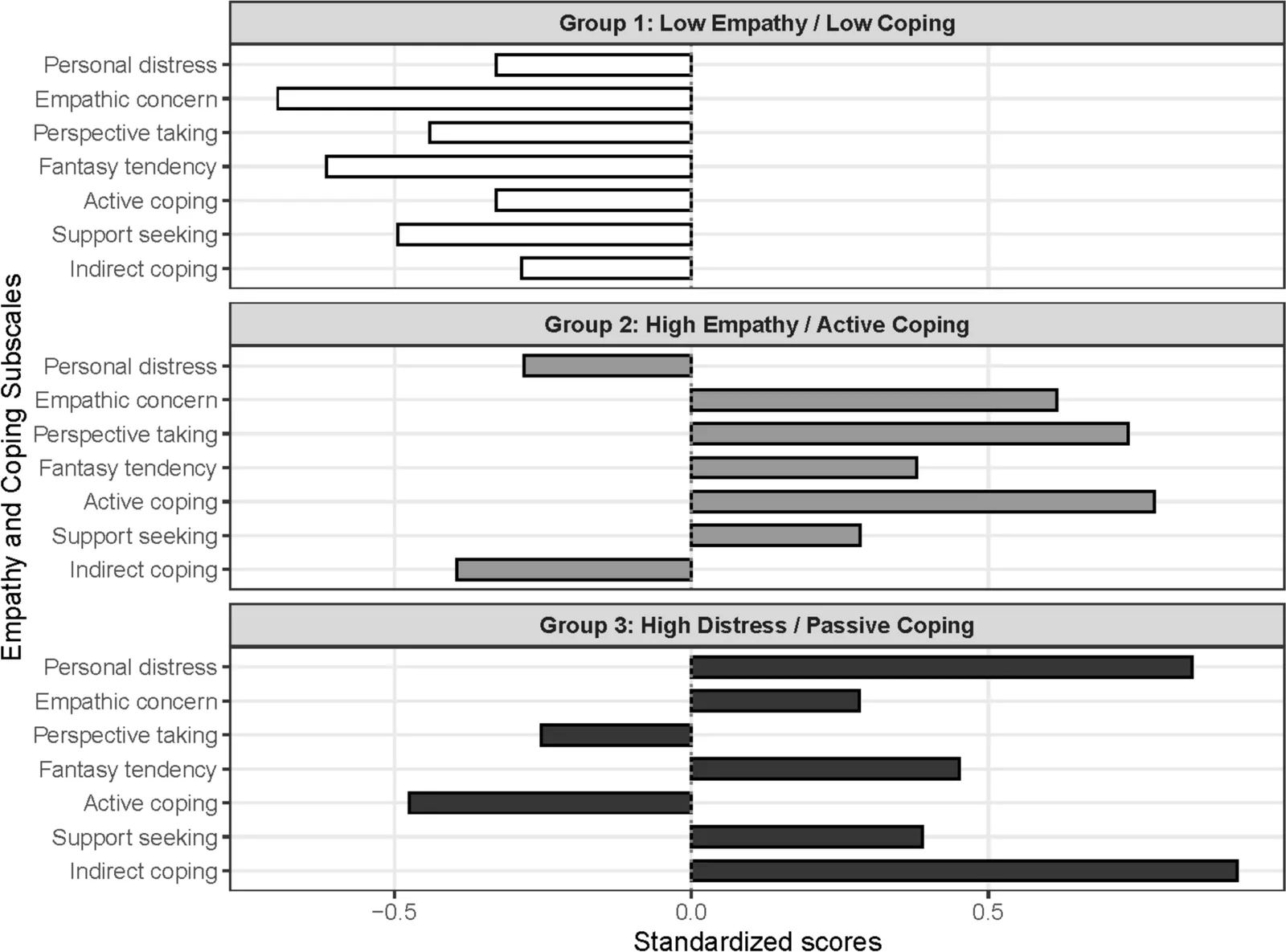
Standardized scores of scales for each group classified by latent profile analysis

LPA using the scores of the four IRI subscales and the three Brief COPE subscales at Time 1 identified three optimal groups based on BIC (−2359.097; model estimation = EII). The groups were: Group 1 (n = 47, 39.8%), Group 2 (n = 38, 32.2%), and Group 3 (n = 33, 28.0%). Figure 1 presents bar charts summarizing the standardized scores for the four empathy and three coping subscales across the latent groups. Group 1 exhibited lower-than-average scores on all IRI and Brief COPE subscales and was labeled the “Low Empathy / Low Coping Group.” Group 2 showed higher-than-average scores in the IRI subscales of empathic concern, perspective taking, and fantasy, with lower-than-average scores in personal distress. Additionally, this group demonstrated higher Brief COPE scores for active coping and support seeking, and lower scores in indirect coping. Based on these characteristics, Group 2 was labeled the “High Empathy / Active Coping Group.” Group 3 had higher-than-average scores in the IRI subscales of personal distress, empathic concern, and fantasy, but lower scores in perspective taking. Further, this group exhibited higher Brief COPE scores for support seeking and indirect coping, while active coping scores were below average. Therefore, Group 3 was labeled the “High Distress / Passive Coping Group.” Table 3 shows the demographic characteristics and average scores for each scale among the three groups at Time 1 and Time 2. No significant group differences were found in the ratio of female to male participants among the three latent groups (*χ*^2^(2) = 2.49, *p* = .29). One-way ANOVA revealed significant differences between the three groups at Time 1 in personal distress, empathic concern, and fantasy (IRI), support seeking and indirect coping (Brief COPE), and STS (ProQOL).

**Table 3:** Demographic characteristics of the three groups and average scores for each scale at Time 1 and Time 2

Measures	Group 1 (Low Empathy / Low Coping Engagement)	Group 2 (High Empathy / Active Coping)	Group 3 (High Distress / Passive Coping)	Test statistic	*p* value	Post-hoc comparisons
N	47	38	33			
Age (mean, SD)	22.0 (1.5)	21.8 (1.3)	21.9 (1.4)	*F* = 0.10	0.91	
Sex (male: female)	12: 35	5: 33	5: 28	*χ*^2^ = 2.5	0.29	
Nursing student (Medical student)	13: 34	7: 31	10: 23	*χ*^2^ = 1.5	0.47	
Interpersonal Reactivity Index: time 1 (mean, SD)						
Personal distress	20.0 (4.4)	20.4 (4.6)	26.4 (4.1)	*F* = 23.7	< 0.001	Group 1 < 3, Group 2 < 3
Empathic concern	21.1 (3.9)	27.2 (2.9)	25.8 (3.3)	*F* = 36.9	< 0.001	Group 1 < 2, Group 1 < 3
Perspective taking	19.9 (3.9)	25.9 (3.9)	21.1 (4.5)	*F* = 25.1	< 0.001	Group 1 < 2, Group 2 > 3
Fantasy tendency	19.9 (4.9)	25.8 (5.8)	26.5 (4.1)	*F* = 22.4	< 0.001	Group 1 < 2, Group 1 < 3
Brief COPE: time 1 (mean, SD)						
Active coping	22.4 (3.0)	26.8 (2.9)	21.9 (2.8)	*F* = 32.4	< 0.001	Group 1 < 2, Group 2 > 3
Support seeking	14.9 (3.2)	18.1 (3.6)	18.5 (3.6)	*F* = 14.0	< 0.001	Group 1 < 2, Group 1 < 3
Indirect coping	26.9 (3.8)	25.7 (3.7)	33.3 (4.3)	*F* = 37.9	< 0.001	Group 1 < 3, Group 2 < 3
ProQOL: time 1 (mean, SD)						
Secondary traumatic stress	17.1 (4.1)	17.7 (3.9)	20.8 (5.4)	*F* = 7.1	0.0012	Group 1 < 3, Group 2 < 3
Compassion satisfaction	27.7 (6.4)	33.2 (5.6)	28.0 (5.5)	*F* = 10.7	< 0.001	Group 1 < 2, Group 2 > 3
Burnout	27.3 (5.3)	23.5 (4.6)	29.4 (4.6)	*F* = 13.3	< 0.001	Group 1 > 2, Group 2 < 3
Interpersonal Reactivity Index: time 2 (mean, SD)						
Personal distress	21.9 (5.0)	20.9 (4.5)	27.1 (3.8)	*F* = 19.2	< 0.001	Group 1 < 3, Group 2 < 3
Empathic concern	20.9 (5.1)	25.7 (3.8)	25.2 (3.6)	*F* = 15.3	< 0.001	Group 1 < 2, Group 1 < 3
Perspective taking	20.7 (4.5)	25.7 (3.8)	21.8 (4.0)	*F* = 16.0	< 0.001	Group 1 < 2, Group 2 > 3
Fantasy tendency	20.6 (5.8)	24.3 (5.9)	26.6 (4.3)	*F* = 12.7	< 0.001	Group 1 < 2, Group 1 < 3
Brief COPE: time 2 (mean, SD)						
Active coping	21.7 (3.1)	26.2 (3.0)	21.2 (3.9)	*F* = 26.3	< 0.001	Group 1 < 2, Group 2 > 3
Support seeking	16.4 (3.7)	18.7 (3.3)	18.4 (3.1)	*F* = 5.7	0.0046	Group 1 < 2, Group 1 < 3
Indirect coping	27.0 (4.0)	26.2 (4.3)	31.7 (4.4)	*F* = 17.5	< 0.001	Group 1 < 3, Group 2 < 3
ProQOL: time 2 (mean, SD)						
Secondary traumatic stress	17.7 (4.7)	18.5 (4.6)	21.6 (4.9)	*F* = 6.8	0.0016	Group 1 < 3, Group 2 < 3
Compassion satisfaction	25.5 (6.1)	31.4 (7.7)	25.3 (6.9)	*F* = 9.8	< 0.001	Group 1 < 2, Group 2 > 3
Burnout	30.0 (5.1)	25.7 (5.5)	33.7 (5.3)	*F* = 20.4	< 0.001	Group 1 > 2, Group 1 < 3, Group 2 < 3

A one-way ANOVA was used to compare continuous variables for the three groups. A chi-square test was used to compare categorical data for the three groups. Post-hoc comparisons were conducted using Tukey's HSD test, and only significant differences (p< .05) were reported

*Brief COPE* brief version of the coping orientation to problems experienced inventory; *proqol*, professional quality of life scale

**Fig. 2:**
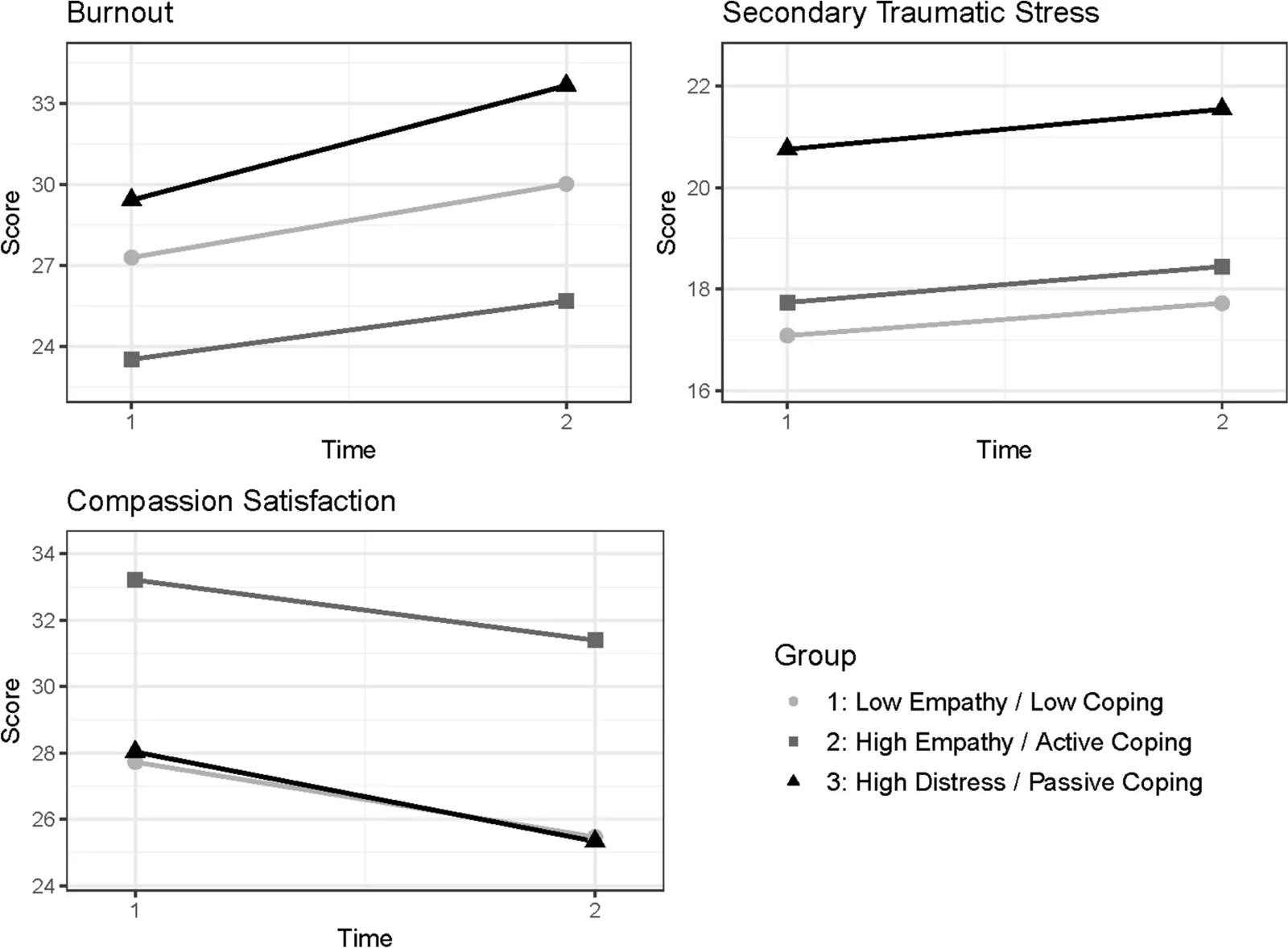
Average changes in psychological responses from Time 1 to Time 2 for each group

Figure 2 illustrates the average changes in psychological responses (compassion satisfaction, burnout, STS) for each group from Time 1 to Time 2. The results of the additional MMRM using LPA-based group classification as fixed effects are shown in Supplementary Table 3. For STS, a significant positive main effect was observed for Group 3 (*t* = 3.3, *p* = .0012) and for gender, with females showing higher levels than males (*t* = 2.0, *p* = .044). For burnout, a significant positive main effect was found for time (*t* = 2.4, *p* = .020), Group 1 (*t* = 4.3, *p* < .001), and Group 3 (*t* = 6.6, *p* < .001). For compassion satisfaction, a significant negative main effect was observed for Group 1 (*t* = -4.6, *p* < .001) and Group 3 (*t* = -4.6, *p* < .001). In addition, both gender and student type showed significant associations, with females showing higher levels than males (*t* = 2.6, *p* = .011) and nursing students showing lower levels than medical students (*t* = − 2.1, *p* = .034). No significant interactions with time were observed for any group.

## Discussion

This study investigated the longitudinal impact of empathy and coping traits on psychological responses among medical and nursing students during their transition from clinical training to clinical practice, aiming to gain insights for developing effective educational interventions related to empathy and coping for healthcare professionals. The results of the MMRM indicated that among empathy traits, personal distress was associated with high STS and burnout as well as low compassion satisfaction, while empathic concern was associated with low burnout and high compassion satisfaction. Regarding coping traits, active coping and support seeking were associated with high compassion satisfaction and low burnout, and indirect coping was associated with high STS and burnout. The LPA classified participants into three groups based on their empathy and coping traits, revealing significant differences in psychological responses among these groups. These findings suggest that variations in empathy and coping traits result in different psychological responses, indicating that educational interventions tailored to individual traits may be effective for medical and nursing students.

Empathic traits, coping strategies, and psychological responses changed significantly over time, suggesting that these factors are influenced by the clinical training process. Specifically, the observed increase in personal distress may reflect the emotional impact of transitioning from training environments to responsible actual patient care. In addition, changes in coping strategies—such as a decline in active coping and an increase in support seeking—may indicate an adaptation to the realities of clinical work, where interpersonal support becomes more vital. The increase in burnout and decrease in compassion satisfaction are consistent with previous studies indicating that burnout increases over time among medical students (Dyrbye et al., 2006; Santen et al., 2010; Schneider et al., 2023), possibly reflecting the harshness of clinical environments, which differ from experiences during training.

Regarding empathy traits, the observed positive effects of empathetic concern—a significant main effect on compassion satisfaction and a protective main effect on burnout—support previous research (Delgado et al., 2023; Shoji et al., 2024), suggesting that it is a critical trait for healthcare professionals. Conversely, personal distress is consistently linked to negative psychological responses in the prior studies (Gleichgerrcht & Decety, 2013; Shoji et al., 2024), including this study, underscoring a need for careful attention as a psychopathological trait. These contrasting associations between the two emotional empathy traits (empathic concern and personal distress) and psychological responses highlight the complexity of empathy as a double-edged sword. Thus, healthcare providers are exposed to both positive and negative psychological reactions associated with empathy. In such situations, they are required to maintain empathy toward patients, which is essential for providing high-quality medical care.

As for coping traits, we confirmed that active coping and support-seeking had positive effects on compassion satisfaction and negative effects on burnout, while indirect coping had positive effects on STS and burnout. Active coping is an essential skill for adapting to stress and challenging situations, and it has been shown to contribute to the mitigation of burnout among healthcare professionals (Wallace et al., 2010). Positive reframing, one of the elements of active coping, has been reported to have a negative correlation with STS and a positive correlation with compassion satisfaction, suggesting protective effects (Măirean, 2016). Similarly, support-seeking strategies have a protective effect on the mental health of healthcare professionals (Koinis et al., 2015). In contrast, indirect coping—particularly denial, substance use, behavioral disengagement, and self-blaming—has been associated with negative psychological outcomes, such as higher levels of secondary traumatic stress and burnout (Akinsulure‐Smith et al., 2018; Chen & Cunradi, 2008). Therefore, strategies that strengthen active coping and support-seeking and reduce indirect coping are considered to be an effective and practical approach.

In the MMRM for psychological responses, we could not find a significant interaction between time and either empathy or coping traits, except for the interaction between time and personal distress on compassion satisfaction. One possible reason is the design of this longitudinal study; the second measurement (Time 2) was conducted approximately one year after medical and nursing students graduated and entered clinical practice. It may have required more time for these various factors to influence their psychological responses. The significant interaction between time and personal distress in compassion satisfaction was contrary to expectations; the group with higher personal distress showed a slower decline in compassion satisfaction from Time 1 to Time 2 compared to the group with lower personal distress. This unexpected result may reflect a “floor effect,” where the compassion satisfaction of those with higher personal distress was sufficiently low at Time 1, which might limit further declines in compassion satisfaction from Time 1 to Time 2.

Adopting a person-centered approach (Howard & Hoffman, 2018), the present study employed LPA to categorize participants into latent groups and compared their psychological responses. Among the three identified groups, Group 2 (High Empathy / Active Coping Group) exhibited the highest levels of empathic concern, perspective-taking, and active coping, along with low levels of personal distress and indirect coping, representing the most favorable empathy and coping traits. This group demonstrated the lowest levels of burnout and the highest levels of compassion satisfaction from Time 1 to 2, reflecting the findings of the variable-centered approach. These results are consistent with a previous cross-sectional study involving 506 healthcare professionals, including medical and nursing students, physicians, and nurses (Shoji et al., 2024), as well as a recent meta-analysis of 21 studies involving medical students (Cairns et al., 2024).

In contrast, Group 3 (High Distress/Passive Coping Group) exhibited the highest levels of personal distress and indirect coping, with low levels of active coping, suggesting that this group possesses less favorable empathy and coping traits. Regarding psychological responses, burnout and STS were the highest in this group across both Time 1 and 2, and compassion satisfaction also showed the lowest scores, which was an expected outcome. These findings are consistent with the results of a cross-sectional study (Shoji et al., 2024) and align with prior research (Thomas, 2013) that demonstrated a relationship between high personal distress and burnout. Meanwhile, Group 1 (Low Empathy/Low Coping Group) showed generally lower scores across most empathy and coping subscales. This group demonstrated the lowest levels of STS but also low compassion satisfaction.

Although the LPA provided a person-centered understanding of the heterogeneous patterns of empathy and coping among medical and nursing students, this approach has several methodological and practical limitations. The profiles reflected distinct baseline in compassion satisfaction, burnout, and STS, and their longitudinal trajectories were broadly parallel. Nevertheless, the profiles highlight meaningful individual differences that are not readily detectable through variable-centered analyses and thus offer an informative framework for understanding heterogeneity in students’ psychological characteristics. Because the findings are preliminary and not yet predictive or directly applicable to educational practice, they should be interpreted with caution. Nonetheless, they suggest that individual differences in empathy and coping may have relevance for future educational considerations.

Thus, considering that psychological responses differ among the three groups classified based on individual empathy and coping traits, educational interventions tailored to these profiles are important and likely to be more effective. In future applications, brief screening tools based on the key empathy and coping subscales that characterize these profiles could be developed to help classify students into similar groups in educational settings. Such tools may support a stratified educational approach in which additional modules are designed to address the specific needs of each group. For example, for groups like Group 1 (Low Empathy/Low Coping Group), which exhibit low empathy and coping traits, educational programs aimed at increasing compassion satisfaction through enhancing empathic concern, active coping, and support seeking are expected to be effective. A meta-analysis of 22 randomized controlled trials evaluating the effectiveness of empathy enhancement programs (Winter et al., 2020) found moderate effects, though no standardized curriculum has been established. Regarding coping education, several studies have examined the effectiveness of stress management education programs in various settings. These interventions have shown positive effects on active coping skills (Hirokawa et al., 2012; Kaveh et al., 2023; Shapiro et al., 2000) and support-seeking skills (Kaveh et al., 2023; Shimazu et al., 2003). Therefore, interventions combining these types of education may be effective.

Meanwhile, for groups like Group 3 (High Distress / Passive Coping Group), which exhibit high levels of personal distress and indirect coping, educational interventions should not only focus on enhancing empathic concern but also on decreasing personal distress and encouraging a shift in coping strategies from indirect to active coping. Adaptive coping strategies such as strengthening social support and setting appropriate boundaries have been shown to be effective in preventing burnout, as demonstrated in a systematic review (Maresca et al., 2022) of seven studies. These elements could also be integrated into educational interventions. In addition, mindfulness and self-compassion-based programs have been reported as effective methods for reducing personal distress and burnout among health care professionals. For instance, a pilot study with nursing students found favorable trends in reducing personal distress following an eight-week mindfulness-based stress reduction program (Beddoe & Murphy, 2004). Similarly, a mixed-methods intervention study with hospital employees demonstrated that a mindfulness-based stress reduction program led to decreases in burnout (Moll et al., 2015). In a study for primary care physicians, mindfulness communication training was associated with reductions in burnout (Krasner et al., 2009). Furthermore, a randomized controlled trial with practicing psychologists showed that mindful self-compassion training reduced stress responses and burnout (Eriksson et al., 2018). These findings suggest that mindfulness and self-compassion-based programs may help mitigate the impact of personal distress and burnout, particularly among those with vulnerable traits.

This study had several limitations. First, the study was conducted with medical and nursing students in Japan, which limits the generalizability of the results. Therefore, the findings should be interpreted with caution. Although the higher proportion of female in our sample primarily reflected the demographic characteristics of the nursing cohort, the proportions of male and female participants did not significantly differ across the LPA-derived groups. In the mixed-effects models, both gender and student type were included as covariates, and adjusting for these variables did not change the direction or significance of the key effects. These findings suggest that gender-related influences are unlikely to have substantially affected the main results, although future studies with more balanced gender distributions would allow a more detailed examination of potential gender-related or cultural influences. Additionally, not all participants provided responses at Time 2, which raises the possibility of attrition bias. Given these limitations, further research with a more diverse sample and complete follow-up data is necessary to confirm the findings.

## Conclusions

This study identified several empathy and coping traits that influence psychological responses during the transition from medical school to clinical practice among medical and nursing students. Additionally, this study demonstrated that differences in the profiles of these traits elicit distinct psychological responses, suggesting that educational interventions tailored to them may yield more effective outcomes. By developing such educational interventions, it may be possible to reduce burnout and STS among healthcare professionals while enhancing compassion satisfaction, thereby enabling the provision of higher-quality healthcare.

## Supplementary Information

Below is the link to the electronic supplementary material.

Supplementary file1 (TIF 32 KB) Average changes in compassion satisfaction from Time 1 to Time 2 across three groups categorized by personal distress levels

Supplementary file2 (DOCX 31 KB)

## Data Availability

The data of this study are available from the corresponding author on reasonable request.
