# Supplementary material for: The Impact of Empathy and Coping Traits on Psychological Responses: A Longitudinal Study on Medical and Nursing Students
Source: J Clin Psychol Med Settings. 2026 Mar 10;33(3):587–97. doi: 10.1007/s10880-026-10144-1 (PMC13582342; doi:10.1007/s10880-026-10144-1)
Supplement: Supplementary file 1 [file 10880_2026_10144_MOESM2_ESM.docx]

Supplementary Table 1. Participants' demographic characteristics between the included and excluded participants

|  | Medical Student | | | | Nursing Student | | | |
| --- | --- | --- | --- | --- | --- | --- | --- | --- |
|  | Included | Excluded | Test statistic | *p* value | Included | Excluded | Test statistic | *p* value |
| N | 30 | 33 |  |  | 88 | 51 |  |  |
| Sex (men: female) | 15:15 | 18:15 | *χ*^2^ = 0.01 | 0.91 | 7:81 | 3:48 |  | 0.75^*^ |
| Age (mean, SD) | 23.9 (0.9) | 24.0 (1.0) | *t* = 0.41 | 0.68 | 21.2 (0.77) | 21.4 (0.8) | *t* = 0.92 | 0.36 |
| Interpersonal Reactivity Index (mean, SD) |  |  |  |  |  |  |  |  |
| Personal distress | 23.0 (6.8) | 21.6 (4.7) | *t* = 0.94 | 0.35 | 21.6 (4.5) | 22.0 (5.1) | *t* = 0.52 | 0.61 |
| Empathic concern | 23.1 (5.7) | 22.9 (4.4) | *t* = 0.12 | 0.90 | 24.8 (3.8) | 24.5 (5.4) | *t* = 0.40 | 0.69 |
| Perspective taking | 21.3 (5.9) | 23.5 (3.5) | *t* = 1.83 | 0.07 | 22.5 (4.4) | 23.1 (4.0) | *t* = 0.87 | 0.39 |
| Fantasy tendency | 23.8 (7.0) | 22.7 (5.0) | *t* = 0.72 | 0.48 | 23.6 (5.5) | 23.4 (5.8) | *t* = 0.17 | 0.87 |
| Brief COPE (mean, SD) |  |  |  |  |  |  |  |  |
| Active coping | 23.5 (3.3) | 23.9 (3.5) | *t* = 0.37 | 0.71 | 23.7 (3.7) | 24.6 (3.6) | *t* = 1.28 | 0.20 |
| Support seeking | 15.6 (3.4) | 15.2 (4.0) | *t* = 0.42 | 0.68 | 17.4 (3.9) | 18.4 (3.8) | *t* = 1.58 | 0.12 |
| Indirect coping | 29.1 (5.1) | 30.5 (7.4) | *t* = 0.89 | 0.38 | 28.1 (5.0) | 28.7 (6.0) | *t* = 0.62 | 0.54 |
| ProQOL (mean, SD) |  |  |  |  |  |  |  |  |
| Secondary traumatic stress | 17.2 (4.6) | 17.1 (4.4) | *t* = 0.04 | 0.97 | 18.7 (4.6) | 18.5 (4.2) | *t* = 0.32 | 0.75 |
| Compassion satisfaction | 30.2 (6.3) | 28.4 (6.3) | *t* = 1.16 | 0.25 | 29.4 (6.4) | 28.6 (7.4) | *t* = 0.63 | 0.53 |
| Burnout | 26.0 (5.5) | 26.5 (4.9) | *t* = 0.41 | 0.68 | 26.9 (5.4) | 27.4 (6.2) | *t* = 0.50 | 0.62 |

Note: An unpaired *t*-test was used to compare continuous data for the two groups. A chi-square test or Fisher's exact test was used to compare categorical data.

*Abbreviation*: Brief COPE, Brief Version of the Coping Orientation to Problems Experienced inventory; ProQOL, Professional Quality of Life scale.

^*^Fisher's exact test

Supplementary Table 2. Pearson's correlation coefficients among scale scores at Time 1 and Time 2

|  | Personal distress | Empathic concern | Perspective taking | Fantasy tendency | Active coping | Support seeking | Indirect coping | Secondary traumatic stress | Compassion satisfaction | Burnout |
| --- | --- | --- | --- | --- | --- | --- | --- | --- | --- | --- |
| Time 1 | | | | | | | | | | |
| Personal distress | 1 |  |  |  |  |  |  |  |  |  |
| Empathic concern | 0.14 | 1 |  |  |  |  |  |  |  |  |
| Perspective taking | -0.17 | 0.36 | 1 |  |  |  |  |  |  |  |
| Fantasy tendency | 0.24 | 0.42 | 0.31 | 1 |  |  |  |  |  |  |
| Active coping | -0.16 | 0.26 | 0.37 | 0.07 | 1 |  |  |  |  |  |
| Support seeking | 0.14 | 0.23 | -0.06 | 0.1 | 0.22 | 1 |  |  |  |  |
| Indirect coping | 0.36 | 0.03 | -0.06 | 0.28 | -0.18 | 0.1 | 1 |  |  |  |
| Secondary traumatic stress | 0.32 | 0.21 | 0.11 | 0.17 | 0.04 | 0.03 | 0.29 | 1 |  |  |
| Compassion satisfaction | -0.23 | 0.27 | 0.15 | 0.21 | 0.42 | 0.17 | -0.15 | 0.04 | 1 |  |
| Burnout | 0.37 | -0.16 | -0.11 | 0.05 | -0.4 | -0.14 | 0.37 | 0.27 | -0.57 | 1 |
| Time 2 | | | | | | | | | | |
| Personal distress | 1 |  |  |  |  |  |  |  |  |  |
| Empathic concern | 0.33 | 1 |  |  |  |  |  |  |  |  |
| Perspective taking | -0.05 | 0.42 | 1 |  |  |  |  |  |  |  |
| Fantasy tendency | 0.41 | 0.53 | 0.25 | 1 |  |  |  |  |  |  |
| Active coping | -0.2 | 0.25 | 0.55 | 0.09 | 1 |  |  |  |  |  |
| Support seeking | 0.14 | 0.47 | 0.27 | 0.27 | 0.27 | 1 |  |  |  |  |
| Indirect coping | 0.38 | 0.07 | 0 | 0.23 | -0.13 | 0.16 | 1 |  |  |  |
| Secondary traumatic stress | 0.54 | 0.28 | 0.06 | 0.33 | -0.06 | 0.17 | 0.47 | 1 |  |  |
| Compassion satisfaction | -0.07 | 0.3 | 0.27 | 0.17 | 0.54 | 0.33 | -0.1 | 0.03 | 1 |  |
| Burnout | 0.49 | -0.05 | -0.18 | 0.14 | -0.5 | -0.13 | 0.37 | 0.44 | -0.67 | 1 |

Note: All correlation coefficients were statistically significant with *p* < 0.001.

Supplementary Table 3. Mixed-effects models for repeated measures with psychological responses as dependent variables

| Dependent variables | Secondary traumatic stress | | | Burnout | | | Compassion satisfaction | | |
| --- | --- | --- | --- | --- | --- | --- | --- | --- | --- |
| Independent variables | Estimate | *t* value | *p* value | Estimate | *t* value | *p* value | Estimate | *t* value | *p* value |
| Time | 1.70 | 1.61 | 0.11 | 2.75 | 2.35 | 0.020 | -2.33 | -1.52 | 0.13 |
| Female (ref: Male) | 2.16 | 2.03 | 0.044 | 0.40 | 0.33 | 0.74 | 3.76 | 2.57 | 0.011 |
| Nursing student (ref: Medical student) | 0.25 | 0.26 | 0.79 | 1.04 | 0.97 | 0.34 | -2.79 | -2.14 | 0.034 |
| Group (ref: Group 2: High Empathy / Active Coping) |  |  |  |  |  |  |  |  |  |
| 1: Low Empathy / Low Coping Engagement | -0.40 | -0.46 | 0.65 | 4.20 | 4.26 | < 0.001 | -5.50 | -4.62 | < 0.001 |
| 3: High Distress / Passive Coping | 3.13 | 3.34 | 0.0012 | 7.10 | 6.60 | < 0.001 | -5.88 | -4.55 | < 0.001 |
| Time * Group 1: Low Empathy / Low Coping Engagement (ref: Group 2: High Empathy / Active Coping) | -0.18 | -0.19 | 0.85 | 0.50 | 0.46 | 0.64 | -0.38 | -0.27 | 0.79 |
| Time * Group 3: High Distress / Passive Coping (ref: Group 2: High Empathy / Active Coping) | -0.07 | -0.06 | 0.95 | 2.00 | 1.70 | 0.092 | -0.81 | -0.52 | 0.60 |
| Time * Nursing student (ref: Medical student) | -1.22 | -1.28 | 0.20 | -0.73 | -0.70 | 0.49 | 0.63 | 0.46 | 0.64 |
| Fit Index |  |  |  |  |  |  |  |  |  |
| Marginal *R*^2^ | 0.15 | | | 0.28 | | | 0.21 | | |
| Conditional *R*^2^ | 0.58 | | | 0.67 | | | 0.58 | | |
